# Synergistic Effects of Ultrasound and Chia Seed Oil on Yak Myofibrillar Protein Gelation: Structure and Gel Property

**DOI:** 10.3390/foods15183214

**Published:** 2026-09-11

**Authors:** Huaifen Wang, Chenyuan Lu, Ying Yang, Yuqi Wang, Lina Wang, Juan Chen, Yuan Liu, Linlin Wang, Rongsheng Du

**Affiliations:** 1 College of Pharmacy and Food, Southwest Minzu University, Chengdu 610041, China; 19881096325@163.com (H.W.); chernwin529@163.com (C.L.); 18130444348@163.com (Y.Y.); wlmdsciggyx@163.com (Y.W.); wangln1025@163.com (L.W.); chenj1221@126.com (J.C.); 13547703273@163.com (Y.L.); 2 Lu’an Soyea Electrical Manufacturing Co., Ltd., Lu’an 237000, China; 3 School of Food and Biological Engineering, Hefei University of Technology, Hefei 230601, China; 4 Sichuan Institute for Drug Control, Chengdu 610016, China

**Keywords:** ultrasound, chia seed oil, minced yak meat, myofibrillar protein, gel properties

## Abstract

The scope of this paper is to investigate the effects of chia seed oil (CSO) ultrasound treatment as a green improvement method for yak myofibrillar protein (MP). We investigated the effects of different concentrations of CSO (0, 1.5, 3.0% *w*/*w*) combined with different ultrasonic power levels (0, 40, 80, 120 W) on the structural and gel properties of yak MP. The CSO-MP system remained stable as an oil-in-water emulsion during testing, with ultrasound serving as the key technique for preparing it. For the moderate ultrasound, CSO significantly increased MP solubility, whiteness, and gel strength, while significantly reducing cooking loss (CL) (*p* < 0.05). Compared with the control group (U0C0), the solubility, whiteness, and gel strength increased by 156.25%, 7.54%, and 89.11%, respectively, while the turbidity, centrifugal loss, and CL decreased by 51.70%, 64.90%, and 32.73%, respectively. Sodium dodecyl sulfate polyacrylamide gel electrophoresis (SDS-PAGE), secondary structure, gel rheological properties, water distribution, and SEM microstructure indicated that moderate ultrasound CSO treatment enhanced the intensity of the myosin heavy chain (MHC) band and the proportion of α-helix and endowed the MP gel with excellent textural properties and water retention. During the ultrasound, CSO (80 W, 3.0%) significantly improved the structural and gel properties of MP.

## 1. Introduction

The yak (*Bos grunniens*), a dominant livestock resource in China’s plateau regions, is rich in various minerals, vitamins, carotenoids, and polyunsaturated fatty acids (PUFAs), holding significant economic and ecological value [1,2]. Compared to ordinary beef, yak meat contains higher levels of protein. However, a study by Guo et al. [3] demonstrated that WHC of cattle was superior to that of yak during postmortem aging, which highlights the need to develop effective processing methods to enhance the WHC of yak meat products. MP, the most influential protein component in muscle tissue, is primarily composed of contractile, regulatory, and scaffold proteins. It significantly affects the sensory attributes, flavor characteristics, and nutritional value of meat products [4]. Moreover, the crucial functional properties of MP, such as emulsification, gelation, and WHC, also substantially influence meat product quality during processing and storage [5]. As a key component of MP, contractile proteins contain MHC, which is vital for MP gel formation and function [6]. Therefore, investigating yak MP is of considerable significance for elucidating the effects of processing on the functional properties of yak meat products.

It is interesting that antioxidant substances (including natural and synthetic) have been widely employed in meat and meat products to extend shelf life and improve functional properties [7]. Among natural antioxidants, plant-derived polyphenolic antioxidants (e.g., essential oils) [8] may scavenge free radicals in protein-based foods by donating hydrogen atoms or electrons via their hydroxyl groups, and may also interact with proteins through non-covalent or covalent bonds to improve functional properties. In particular, gelation properties, the most important textural attribute of processed muscle foods, are susceptible to such interactions [9]. Numerous studies have demonstrated that the diverse active components in plant essential oils possess excellent free radical scavenging capacity and are safer than synthetic. For instance, cinnamon essential oil, clove essential oil, and lemon essential oil have been shown to exert strong antimicrobial and antioxidant activities during meat processing, thereby improving the functional properties of meat products [10,11,12].

Chia seeds, rich in protein, carbohydrates, and dietary fiber, possess anti-inflammatory, blood sugar regulatory, and antioxidant properties [13]. CSO extracted from them is a novel functional food ingredient rich in PUFAs, uniquely characterized by its high content of *ω*-3-linolenic acid and other effective components; related studies indicate that components in CSO, such as flavonoids, polyphenols, phytosterols, and tocopherols, may possess potential effects such as antioxidant activity, immune enhancement, and alleviation of cognitive decline [14,15,16].

Ultrasound, as an emerging non-thermal food processing technology, converts electrical energy into high-frequency mechanical energy, altering the physical and chemical properties of foods. Several studies have shown that the cavitation effect of ultrasound can modify muscle molecular structure, improve marination rates, and enhance meat tenderness. For instance, Shi et al. [17] evaluated the impact of ultrasound on MP structure and gel properties, finding that treating chicken breast MP with added potassium alginate significantly improved its WHC and gel structure. Regarding combination with CSO, Aryasree et al. [18] utilized ultrasonic emulsification to develop CSO monolayer and multilayer emulsions via electrostatic deposition, concluding that monolayer emulsions had the highest stability and multilayer emulsion-based powders had better flowability. These findings suggest ultrasound’s potential for improving edible quality, water retention, and physicochemical properties of meat products. Generally, ultrasonic cavitation breaks down macromolecular polyunsaturated oils into micron/nano-sized oil droplets, which greatly enhances the contact specific surface area between the oil and protein phases, thereby reducing the resistance to hydrophobic binding. Moreover, the transient high-pressure shock waves and microjet shear generated during ultrasonication might weaken the spontaneous hydrophobic aggregation tendency among MP molecules and inhibit the self-assembly of myosin heavy chains [19]. The free and unfolded MP molecules might actively migrate to the oil–water interface and undergo molecular-level interactions with the oil. Compared with saturated oils, CSO is rich in PUFAs, whose carbon chains possess greater flexibility, thereby increasing the likelihood of their insertion into the hydrophobic cavities or grooves of MP. This facilitates the formation of multi-layered van der Waals synergistic interactions and hydrophobic coupling effects, thereby enhancing the stability of the MP system [20]. Furthermore, the natural conformation of yak MP is more loosely structured, resulting in a higher degree of exposure of hydrophobic residues. Ultrasound treatment might further provide additional hydrophobic binding sites for CSO, endowing yak MP and CSO with stronger potential for hydrophobic interactions [3]. Currently, research on the mechanisms underlying changes in the structure, function, and rheological properties of yak meat MP after ultrasound treatment is limited. Furthermore, the application of CSO in meat products remains relatively simplistic. Existing research on CSO primarily focuses on its own physico-chemical bioactive effects [21,22], with few studies investigating its impact on MP in meat products and its mechanisms. Research on the combined effect of CSO and ultrasound treatment on the functional properties of yak meat MP and its mechanism has not been reported and warrants in-depth exploration. Therefore, this study employed gradient concentrations of CSO combined with different ultrasonic power intensities to investigate their effects and mechanisms on the structural and gel properties of yak meat MP.

The study aims to deeply investigate the mechanisms by which combined CSO and ultrasound treatment affect the structural properties (solubility, turbidity, secondary structure, SDS-PAGE) and gel functional properties (rheology, microstructure, water distribution, etc.) of yak meat MP. It seeks to elucidate the specific mechanisms through which this combined treatment improves the gel functional properties of yak meat MP, while identifying the optimal treatment parameters. The findings are expected to provide a novel approach for enhancing the functional properties of MP in yak meat batter products. This research not only provides a certain theoretical reference for enhancing the functional properties of yak meat products and thereby improving their overall quality, but also offers theoretical support for the efficient and high-value utilization of CSO in yak meat and meat products.

## 2. Materials and Methods

### 2.1. Materials

The yak meat samples required for this experiment were sourced from four male yaks (approximately 4 years old, in good growth condition, and with similar body weights). The longissimus dorsi muscles were obtained from Xiangyang Jiangnan Slaughterhouse (Guanghan, China). After removing surface fat and connective tissue, the muscles were evenly cut into approximately 80.0 g pieces along the myofibril direction and pooled for subsequent use. All indicator measurements were completed within 24 h (the limitation of this study is the failure to record and measure postmortem aging time and ultimate pH of the muscle). The CSO (98% purity) was purchased from Minglang Biotechnology Co., Ltd. (Xi’an, China), and all other analytical-grade chemicals were provided by Kelong Chemical Co., Ltd. (Chengdu, China).

### 2.2. MP Extraction and Sample Preparation

#### 2.2.1. Extraction of MP

After MP extraction following the method of Huang et al. [23], the concentration was determined using the biuret method with bovine serum albumin (BSA) as the standard on a UV-1900 ultraviolet spectrophotometer (Aoyi Instrument (Shanghai) Co., Ltd., Shanghai, China) [24]. The resulting samples were stored at 4.0 °C and the relevant indicators were measured within 48 h.

#### 2.2.2. Preparation and Ultrasound Treatment of the CSO-MP System

Following the method of Zhang et al. [25] with slight modifications, MP was diluted to 30.0 mg/mL using 15.0 mmol/L phosphate-buffered saline (PBS) containing 0.6 mmol/L NaCl (pH 6.25). Then, 300 mL of the MP solution (in a 500 mL beaker) was transferred to a jacketed beaker with circulating ice water. CSO was added to the MP dispersion at concentrations of 0, 1.5, and 3.0% (*w*/*w*), designated as C0, C1, and C2, respectively. The CSO-MP system was subjected to ultrasound treatment for 8 min using a SCIENTZ-IID ultrasonic cell disruptor (Xinzhi Biotechnology Co., Ltd., Ningbo, China) equipped with a 20 mm probe immersed 2.0 cm from the bottom of the liquid. The sample temperature was maintained at 10 ± 0.2 °C. The working mode was set to 2 s on and 2 s off, with power levels of 0, 40, 80, and 120 W, designated as U0, U1, U2, and U3, respectively. The resulting combinations were designated as U0C0, U0C1, U0C2; U1C0, U1C1, U1C2; U2C0, U2C1, U2C2; and U3C0, U3C1, U3C2, for a total of 12 groups. U0C0 served as the blank control group without ultrasound treatment and without CSO addition.

#### 2.2.3. Thermally Induced Gelation of the CSO-MP System

Freshly extracted 30.0 mg/mL MP-CSO dispersion (within 24 h) was transferred to 50 mL centrifuge tubes. The samples were heated in a water bath from 20.0 °C to 80.0 °C at a controlled rate, held at 80.0 °C for 30 min, immediately cooled in an ice–water mixture, and then stored at 4.0 °C for 12 h before subsequent analyses.

### 2.3. Parameters for MP Structural Properties

#### 2.3.1. MP’s Solubility and Turbidity

The methods of Du et al. [26] were followed with slight modifications. MP was prepared as a 2.0 mg/mL suspension and allowed to stand at 4.0 °C for 1 h. The suspension was then centrifuged at 2000× *g* for 20 min using a 5804R high-speed refrigerated centrifuge (Eppendorf, Hamburg, Germany). The supernatant was collected, and its protein concentration was determined using the biuret method. Solubility was calculated as the ratio of the protein concentration in the supernatant after centrifugation to that before centrifugation, multiplied by 100%. For turbidity measurement, PBS (containing 0.6 mmol/L NaCl, pH 6.25) was used as the blank, and the absorbance was measured at 600 nm.

#### 2.3.2. SDS-PAGE

The method of Chen et al. [27] was employed with minor adjustments. MP concentration was adjusted to 2.0 mg/mL. For each sample, one aliquot was mixed with 4× non-reducing sample buffer (non-reduced condition), and another aliquot was mixed with 40.0 µL of 10% β-mercaptoethanol (reduced condition). The mixtures were heated in a boiling water bath for 5 min and cooled to room temperature before loading (10.0 µL per well). Electrophoresis was performed using a DYY-6C electrophoresis system (Liuyi Biotechnology Co., Ltd., Beijing, China) at a constant voltage of 80 V. Gels were stained with Coomassie Brilliant Blue R-250 for 1 h and destained until a clear background was achieved before imaging.

#### 2.3.3. Secondary Structure

Following the method of Wang et al. [28] and Pan et al. [9] with slight modifications, freeze-dried powders containing 30.0 mg/mL CSO-MP and CSO alone (prepared at volumes corresponding to 1.5% and 3% of MP) were prepared. Each sample was mixed with dried potassium bromide (KBr) at a mass ratio of 1:200 and compressed into pellets. Full-spectrum data were collected using an LR 64912C Fourier transform infrared spectrometer (PerkinElmer, Waltham, MA, USA) over a scanning range of 4000–400 cm^−1^ at a resolution of 5 cm^−1^, with 16 scans per sample. All original MP spectra were processed using OMNIC 9.2 software. Baseline correction was performed first, followed by spectral subtraction using the CSO reference spectrum. The subtraction factor was continuously adjusted until the characteristic ester carbonyl absorption peak at 1720–1740 cm^−1^ completely disappeared, thereby eliminating the interference of lipid signals on the amide I band (1600–1700 cm^−1^). Using PeakFit 4.0 software with the Gaussian peak fitting algorithm, the lipid-subtracted spectra were sequentially subjected to smoothing, Fourier self-deconvolution, and second derivative transformation to quantitatively determine the proportions of each protein secondary structure.

### 2.4. Parameters for MP Gel Functional Properties

#### 2.4.1. Rheological Properties of Gels

Following the method of Du et al. [29] with modifications, a 2.0 mL aliquot of the sample was loaded, and the edges of the parallel plates were sealed with silicone oil to prevent moisture evaporation. Temperature sweep tests were performed using a DISCOVERY HR-1 rotational rheometer (TA Instruments, New Castle, DE, USA) with parallel plates. The temperature was increased from 25.0 °C to 85.0 °C at a rate of 2.0 °C/min, The gel was subjected to continuous oscillatory shear mode at a fixed frequency of 0.1 Hz and a controlled strain of 0.5%, with a plate gap of 2000.0 μm.

#### 2.4.2. Gel Whiteness

The surface moisture of MP gels was blotted dry with filter paper. The color parameters (L*, a*, b*) were measured using a CR-400 colorimeter (Konica Minolta, Tokyo, Japan). Parameter settings: D65 standard illuminant, 2° standard observer; aperture diameter 8 mm, illumination area 11 mm. Whiteness was calculated according to the formula described by Du et al. [26].

#### 2.4.3. Gel Strength

The method of Gao et al. [30] was followed with slight modifications. MP solid gel samples were cut into cylinders of approximately 20 mm in height. Gel strength was analyzed at room temperature (25 ± 2 °C) using a TA.XT. Plus a texture analyzer (Stable Micro Systems, Godalming, Surrey, UK) equipped with a cylindrical probe (P/0.5, diameter 12.0 mm). The test speed was 1.0 mm/s, the penetration distance was 10.0 mm, and the trigger force was 5.0 g, each sample was measured in six replicates.

#### 2.4.4. CL of the Gels

The method of Diao et al. [31] was adapted. The mass of the MP solution before heating (*m*_1_) was recorded. Samples were heated in a water bath (HH-6, Guohua Instrument Manufacturing Co., Ltd., Changzhou, China) from 20.0 °C to 80.0 °C and held for 30 min. After cooling to room temperature, gels were refrigerated at 4.0 °C overnight. Surface moisture was removed, and the mass (*m*_2_) was recorded. CL (%) was calculated according to Equation (1).

(1) $$CL=\frac{m_{1}-m_{2}}{m_{1}}\times 100\%$$

#### 2.4.5. Centrifugal Loss of the Gels

The procedure of Wang et al. [32] was modified. A known mass of gel was placed in a 50 mL centrifuge tube and centrifuged at 3000× *g* for 15 min at 4 °C using a 5810 R high-speed refrigerated centrifuge (Eppendorf, Hamburg, Germany). The masses of the empty tube (*m*_0_), the tube with gel before centrifugation (*m*_1_), and after centrifugation (*m*_2_) were recorded. Centrifugal loss (%) was calculated according to Equation (2).

(2) $$Centrifugal\;loss=\frac{m_{2}-m_{0}}{m_{1}-m_{0}}\times 100\%$$

#### 2.4.6. Low Field Nuclear Magnetic Resonance (LF-NMR) Analysis

Transverse relaxation time (*T*_2_) was determined at 32.0 °C using a Carr–Purcell–Meiboom–Gill sequence as reported by Pereira et al. [33]. The parameters were set as follows: repetition time of 4000 ms, 10,000 echoes, and 8 scans. Raw data were displayed as transverse relaxation decay curves, and the *T*_2_ values, corresponding peak areas (*A*_2_), subsequently, their proportional peak fractions (*P*_2_) were calculated.

#### 2.4.7. Gel Microstructure

Following the method of Han et al. [34], gel samples were cut into small cubes (2.0 × 2.0 × 2.0 mm), freeze-dried for 18 h using a D-37520 freeze-dryer (Martin Christ, Osterode am Harz, Germany), and sputter-coated with gold. The microstructure was observed using GeminiSEM360 scanning electron microscope (Carl Zeiss AG, Baden-Württemberg, Germany) at an accelerating voltage of 3.00 kV and a magnification of 300×.

### 2.5. Statistical Analysis

Prior to the experiment, the longissimus dorsi muscle samples (approximately 80.0 g per portion) obtained from the four yaks were thoroughly pooled to eliminate inherent background differences among individual animals. Subsamples were then randomly drawn from the pooled meat for subsequent experimental treatments, and each indicator was measured in triplicate as technical replicates. All statistical analyses in this study were performed based on data derived from these technical replicates. Data are presented as mean ± standard deviation in Microsoft Excel (version 2021). Significance of differences among means was analyzed using Duncan’s multiple range test in SPSS software (version 27.0). Pearson correlation coefficients between different parameters were calculated, and cluster analysis was performed using R software (version 4.4.3) with RStudio. A probability value of *p* < 0.05 was considered statistically significant. All other graphs were plotted using Origin software (version 2021).

## 3. Results and Discussion

### 3.1. Combined Effect of CSO and Ultrasound on MP Structure

#### 3.1.1. Effects of CSO with Ultrasound on the Solubility and Turbidity of MP

**Figure 1:**
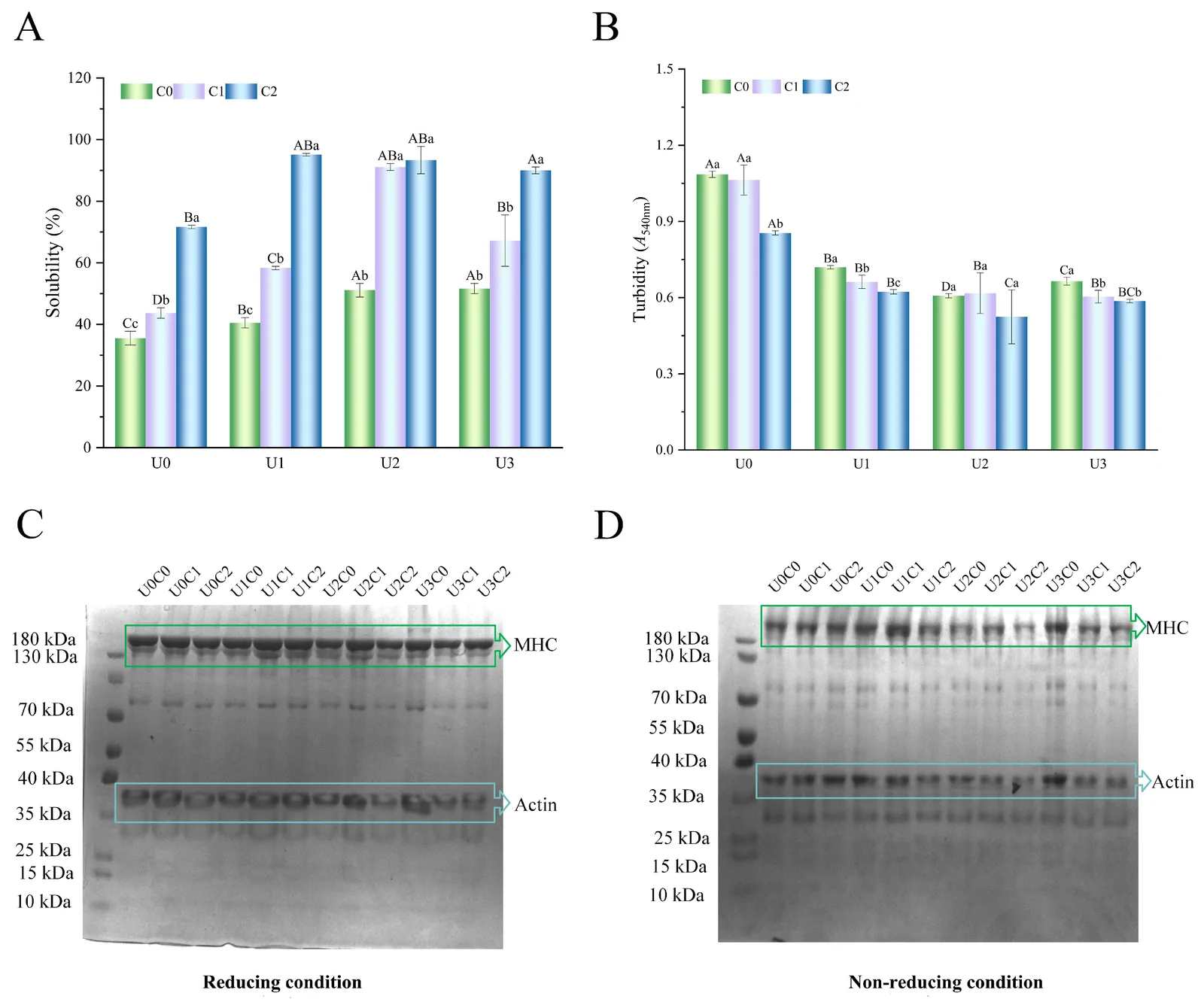
Effects of different concentrations of CSO combined with different ultrasonic power levels on the (**A**) solubility, (**B**) turbidity, and SDS-PAGE patterns under (**C**) reduced and (**D**) non-reduced conditions (indicating cross-linking degree) of yak meat MP. CSO concentrations: 0% (C0), 1.5% (C1), and 3.0% (C2). Ultrasonic power: 0W (U0), 40W (U1), 80W (U2), 120W (U3). Combined treatments are denoted as U0C0, U0C1, U0C2; U1C0, U1C1, U1C2; U2C0, U2C1, U2C2; U3C0, U3C1, U3C2 (12 groups in total). Differences were analyzed using Duncan’s new multiple range test. Different lowercase letters above bars indicate significant differences (*p* < 0.05) among different CSO treatment groups at the same ultrasonic intensity. Different uppercase letters indicate significant differences (*p* < 0.05) among different ultrasonic intensities within the same CSO treatment group. All measurements were performed in triplicate as technical replicates, with samples randomly drawn from the pooled longissimus dorsi muscle obtained from four yaks. Error bars represent the standard error of the mean. The same conventions apply to the following figures.

Solubility and turbidity are fundamental functional properties of MP that directly reflect the extents of denaturation and aggregation of MP, where higher turbidity indicated more pronounced protein aggregation. As shown in Figure 1A, the solubility of MP increased significantly with increasing elevated CSO concentration and ultrasonic power (*p* < 0.05). The U1C2 group achieved the maximum solubility at 95.11%, which was significantly greater than the 35.56% recorded in the U0C0 group (*p* < 0.05). At the same ultrasonic power, MP solubility showed a significant upward trend with increasing CSO concentration (*p* < 0.05), displaying a certain concentration dependence. When the addition ratio of CSO was fixed, the solubility of MP showed a significant increasing trend with the increase in ultrasonic power compared to the C0U0 group (*p* < 0.05). CSO, as a natural amphiphilic molecule, may spontaneously accumulate at the oil–water interface, thereby modulating the interfacial arrangement of MP molecules and the interfacial tension of the system, and facilitating the uniform spreading of MP on the surface of CSO droplets. Furthermore, appropriate ultrasonic cavitation and mechanical shear may remodel water–MP interactions and the aggregated structure of macromolecules, as well as enhance intermolecular electrostatic repulsion, thereby enabling the dissociation of dense protein aggregates and inhibiting the re-aggregation and precipitation of proteins [35], ultimately leading to a substantial improvement in MP solubility. Meanwhile, ultrasonic cavitation tends to generate •OH radicals, but abundant polyphenols, flavonoids and other substances in CSO may alleviate MP aggregation [18], and prevent random cleavage of peptide chains, thereby improving protein solubility to a certain extent [19]. Nevertheless, as ultrasonic power further increases, solubility exhibits a slight downward trend, although this difference is not statistically significant (*p* > 0.05). It is hypothesized that excessive ultrasound treatment may cause potential detrimental structural damage to MP. This aligns with the findings of Sun et al. [36] regarding the impact of ultrasound treatment on the functional properties of carp MP.

As illustrated in Figure 1B, turbidity values of all treated groups presented an overall decreasing trend compared to U0C0, and the U2C2 group possessed the minimum turbidity. With the gradual elevation of ultrasonic power, turbidity for all groups dropped significantly at first and then rose (*p* < 0.05), and MP exhibited the lowest turbidity under U2 (80 W) ultrasound treatment. Specifically, compared with the U0C0 group, the turbidity of the U2C0, U2C1 and U2C2 treatments declined by 44.05%, 43.13% and 51.70%, respectively. Combined with the above solubility data, it is hypothesized that moderate ultrasound combined with CSO may effectively alleviate protein aggregation and strengthen water–MP interactions, thereby stabilizing the MP system. Ultrasonic processing may improve the hydrophilicity and dispersibility of MP. Meanwhile, it may facilitate the formation of finer, more homogeneous CSO emulsion droplets that occupy hydrophobic cavities within MP molecules. These observations were consistent with the research outcomes reported by Jambrak [37]. Nevertheless, as the ultrasonic power further increased to 120 W, the turbidity showed an upward trend (*p* > 0.05), which was similar to the solubility results shown in Figure 1A. Over-intensive ultrasound treatment may exert detrimental impacts on the MP system, triggering severe protein unfolding, as well as intensifying intermolecular cross-linking and aggregation. In addition, CSO is rich in PUFAs bearing multiple carbon–carbon double bonds, rendering these PUFAs highly susceptible to oxidative attack under harsh external conditions. High-power ultrasound treatment at 120 W may induce drastic acoustic cavitation within the MP–CSO mixed matrix. Instantaneous extreme high temperature and pressure generated during cavitation-bubble collapse may accelerate severe lipid peroxidation of PUFAs in CSO [38]. Accordingly, continuous generation of lipid peroxyl radicals, alkoxyl radicals and secondary lipid oxidation products may occur under U3 treatment. These reactive species may initiate cross-linked CSO–MP co-oxidation, which may disrupt MP molecular conformation and induce various irreversible structural damage to proteins. Furthermore, such oxidation reactions may cause abnormal protein aggregation and polypeptide fragmentation [39], ultimately elevating MP turbidity and reducing protein solubility.

#### 3.1.2. Effects of CSO with Ultrasound on the SDS-PAGE of MP

Two types (reduced and non-reduced) of SDS-PAGE were adopted to investigate the combined influences of varying CSO dosages and ultrasonic powers on the composition, cross-linking extent and polymerization degree of MP. The separation molecular weight marker covered a range of 10–180 kDa. Figure 1C,D display the SDS-PAGE band patterns (reduced and non-reduced) that primarily contained the *MHC* (~180 kDa) and actin (~40 kDa), respectively. As observed in the reduced electrophoresis gel (Figure 1C), the band intensity of the MHC region first increased and subsequently declined with elevated CSO dosage and ultrasonic power. Meanwhile, the band intensity of proteins at approximately 70 kDa continuously decreased. Overall, the U1C1 group exhibited the optimal protein distribution state. It is hypothesized that cavitation effects triggered by elevated ultrasonic power may rearrange MP molecular structures and facilitate the migration of various protein fractions towards MHC (around 180 kDa). Moreover, under high-shear ultrasonic force, gradually supplemented CSO may form nano- and micro-sized emulsion droplets acting as rigid active fillers; these particles may suppress MP degradation triggered by cavitation-derived free-radical oxidation. Additionally, these droplets may drive low-molecular-weight proteins to bind to stable MHC, which may optimize MP composition and preserve the structural integrity of protein fractions. Nevertheless, when ultrasonic power was further increased, the band intensity in the MHC region gradually diminished, whereas the intensity in the actin region rose. Combined with the data presented in Figure 1A,B, high-power ultrasound may trigger oxidation-driven cross-linking of sulfhydryl-rich actin, darkening actin bands, lowering protein solubility and elevating turbidity synchronously. Meanwhile, ultrasonic cavitation may disperse MP aggregates and depolymerize macromolecular polymers [40], which may alter MP molecular conformation. Furthermore, although direct oxidation indicators were not determined in the present study, CSO, which is abundant in PUFAs, exhibits high chemical lability and is extremely prone to oxidative damage. It is hypothesized that high-power ultrasound treatment at 120 W may provide sufficient activation energy to trigger severe lipid peroxidation, and the resultant lipid radicals may attack reactive amino acid residues of MP, including tyrosine, cysteine and tryptophan [41]. This oxidative process may cause irreversible structural damage to MP, including disulfide bond cleavage, disruption of ordered secondary structures (e.g., α-helices), and the occurrence of abnormal protein aggregation and polypeptide fragmentation. This observation aligned with the findings of Wang et al. [42], who investigated how high-power ultrasound altered the structural and functional properties of yellow croaker MP.

Non-reduced SDS-PAGE retains intrinsic disulfide bonds within MP, which allows direct characterization of protein polymerization under native-like conditions. As displayed in the non-reduced gel (Figure 1D), CSO synergized with moderate ultrasound treatment to facilitate disulfide cross-linking, gradually intensifying MHC bands as CSO dosage and ultrasonic power increased. Simultaneously, the actin band intensity declined as the CSO supplementation ratio rose. It is hypothesized that CSO supplementation may stabilize high-molecular-weight protein complexes and alleviate gel smearing originating from the cross-linking and aggregation of sulfhydryl-rich actin. Nevertheless, when ultrasonic power elevated to 120 W, the actin band intensity gradually increased, consistent with the band characteristics observed in reduced gels. In summary, a comprehensive analysis of Figure 1A–D hypothesized that the synergistic combination of ultrasound and CSO may optimize the composition of yak meat MP by modulating disulfide bonds and hydrophobic interactions. This synergism may improve the overall structural properties of yak MP. By contrast, excessive ultrasonic power coupled with high-dose CSO may trigger oxidative cross-linking of sulfhydryl-rich actin and accelerate MP depolymerization, which may exert detrimental impacts on MP structural stability.

#### 3.1.3. Effects of CSO with Ultrasound on the Secondary Structure of MP

**Figure 2:**
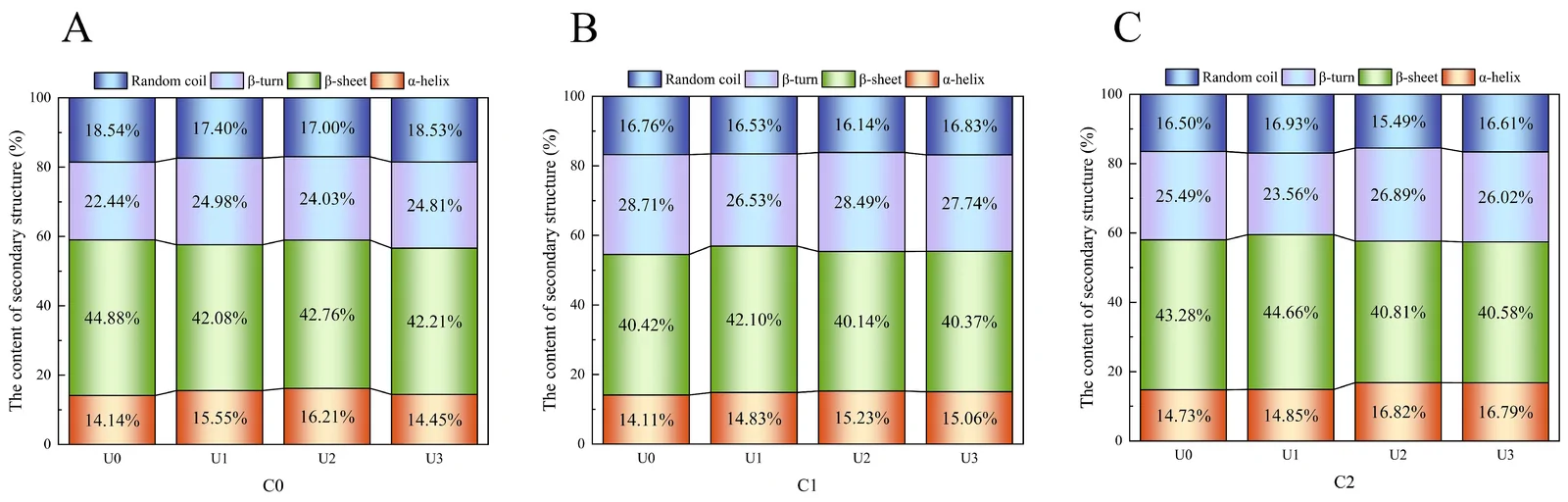
Effects of different concentrations of CSO combined with different ultrasonic power levels on (**A**–**C**) the secondary structure of yak meat MP.

Figure 2A,B,C, respectively, illustrate the variations in secondary structural fractions of yak meat batter MP from yak meat batter treated with different ultrasonic powers and CSO concentrations. Compared to the untreated U0C0 control group, as the ultrasonic power and CSO concentration increased, the overall α-helix proportion of all groups gradually increased, whereas the random coil fractions decreased. The U2C2 group possessed the most ordered protein conformation, with the maximum α-helix fractions reaching 16.82%. At a fixed CSO supplementation level, α-helix fractions displayed an overall upward trend as ultrasonic power increased. Kim et al. [40] reported that ultrasound treatment increased the α-helix proportions of porcine MP. Zhu et al. [43] reported that ultrasound treatment promoted an increase in the α-helix content of chicken MP from 20% to 37%. Xie et al. [31] found that ultrasound-assisted heating triggered conformational transitions of silver carp MP from random coils to α-helices and β-sheets. The authors proposed that ultrasonic shear reshaped MP secondary structures and reconstructed intramolecular hydrogen bonds within disordered peptide chains, converting disordered fragments into ordered α-helical structures. Consistent tendencies were detected in the current experiment. Moderate ultrasound may have facilitated hydrogen-bond formation in MP and accelerated the transformation of random coils to α-helices, strengthening MP structural stability. Nevertheless, several previous investigations have recorded contradictory structural variations: reduced α-helix proportions coupled with elevated fractions of β-sheets and random coils. Such inconsistent observations may stem from divergent ultrasonic parameters and distinct protein matrix compositions across different research systems [44]. CSO contains abundant hydrophobic alkyl chains and polar carboxyl groups. Its intrinsic amphipathic structure may enable hydrophobic interactions and hydrogen bonding with protein molecules. During ultrasound treatment, CSO may also spontaneously be inserted into MP hydrophobic cavities, rearranging intramolecular non-covalent forces, stabilizing MP molecular conformation and providing structural protection for proteins. Meanwhile, combined ultrasonic processing emulsified CSO into nanoscale oil droplets. These tiny particles may physically separate MP molecules and suppress irreversible protein aggregation [16]. Prior research confirmed that CSO contains substances such as vitamin E, phytosterols and polyphenols, which implies that it may also mitigate structural damage induced by free radicals generated during ultrasonic cavitation [15]. Combined with the foregoing solubility, turbidity and SDS-PAGE data, the above conformational variations may arise from ultrasonic mechanical cavitation. Cavitation may reshape and optimize MP structures, improve the dispersion of CSO throughout the protein matrix, and intensify molecular interactions between CSO and MP. These intermolecular interactions may further stimulate hydrogen-bond generation and elevate the relative proportion of α-helical conformations. These ordered structural features may improve MP solubility, lower system turbidity, and restrain excessive aggregation and degradation of proteins, which may corroborate all previous experimental results. Nevertheless, notable structural deterioration was observed at elevated ultrasonic power and CSO dosage: the C2U3 group (high CSO concentration coupled with 120 W ultrasound) displayed a sharp rise in random-coil fractions. This phenomenon may originate from intense cavitation generated by high-power ultrasound, which may have triggered excessive unfolding of MP polypeptide chains. In addition, CSO is rich in polyunsaturated fatty acids (PUFAs), which may readily undergo oxidative reactions under high-power ultrasound treatment [14]. Lipid oxidation may further induce severe protein unfolding and intermolecular aggregation, hindering the construction of uniform gel networks and damaging the processing properties of yak meat protein gels.

### 3.2. Impact of CSO Ultrasound Treatment on Gel Function in Yak Meat MP

#### 3.2.1. Effects of CSO with Ultrasound on Rheological Properties of MP Gels

**Figure 3:**
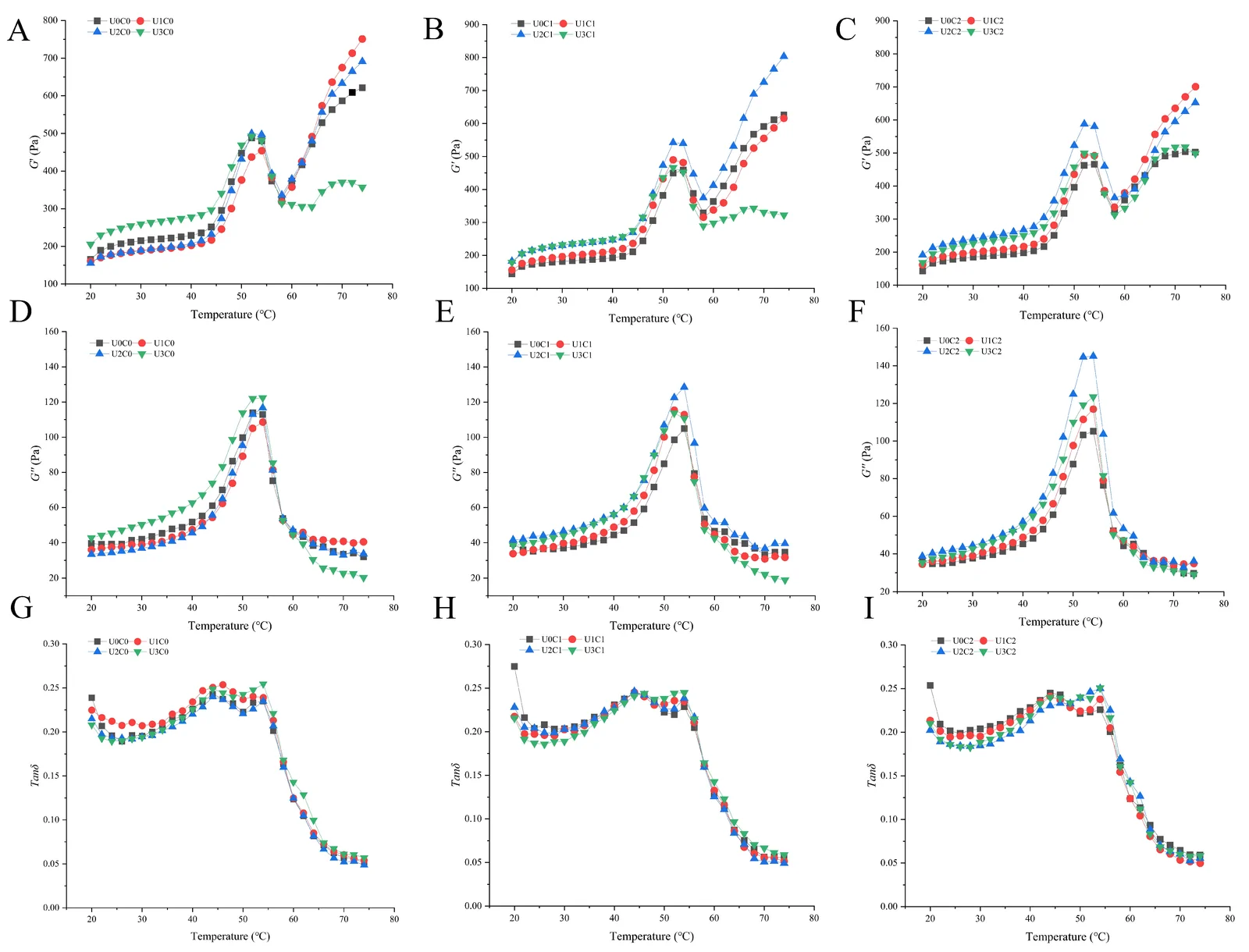
Effects of different concentrations of CSO combined with different ultrasonic power levels on the (**A**–**C**) storage modulus (*G*′), (**D**–**F**) loss modulus (*G*″), and (**G**–**I**) loss tangent (*tan δ*) of yak meat MP gels during thermal gelation.

Figure 3A–I display rheological variations (storage modulus (*G*′), loss modulus (*G*″), and loss tangent (*tan* *δ*)) throughout gelation of MP extracted from yak meat batter, after treatments with various ultrasonic powers and CSO concentrations. As shown in Figure 3A–C, *G*′ increased sharply within the temperature range of 40–52 °C for all groups, a phase where myosin heads were cross-linked, and the gel network began to form. As the temperature further elevated to 52–60 °C, *G*′ declined, which may be attributed to the disruption of nascent gel networks induced by myosin–actin interactions [45]. Subsequently, with further temperature increase, *G*′ exhibited a significant overall surge. Abundant hydrophobic interactions and disulfide bonds may form within this temperature window, converting the system from a viscous liquid into an elastic solid gel matrix. For C0 samples, peak G′ values increased with rising ultrasonic power. It is hypothesized that ultrasound may endow MP gels with stronger elastic properties. At the same ultrasonic power, incremental CSO addition also led to an overall increase in *G*′. It is hypothesized that high-power ultrasound may break CSO into fine droplets, with amphiphilic MP spontaneously adsorbing at the oil-water interface. This treatment may promote interactions between MP-MP and MP–water, facilitating moderate cross-linking and the formation of a uniform gel network, thereby significantly enhancing *G*′. This observation aligned with the findings of Wang et al. [46]. Nevertheless, under 120 W ultrasonic power, all treatments exhibited substantially lower final *G*′ values relative to the U0 (0 W) control and other groups. Meanwhile, *G*′ decreased abnormally with increasing CSO dosage for the U0 group compared with all other test groups. It is hypothesized that this reduction arises from extreme cavitation induced by excessive ultrasonic power, which may have triggered severe MP denaturation and broken the chemical bonds that maintain the three-dimensional gel network structure. As a consequence, the gel network may collapse and lose its elastic capacity. Combined with the foregoing characterization data, this deterioration may also stem from intense cavitation under high-power ultrasound, which may have accelerated peroxidation of PUFAs abundant in CSO. Lipid peroxidation may then trigger substantial polypeptide unfolding and spontaneous protein aggregation, destabilizing the three-dimensional gel network and diminishing the elastic performance of MP gels [47]. Such structural damage may prevent the formation of homogeneous, compact elastic gel frameworks and reduce *G*′. In brief, the combination of appropriate CSO supplementation and mild ultrasound may exert a synergistic effect. The resulting gels may show superior elasticity upon external loading, accompanied by elevated *G*′ and facilitated assembly of compact elastic gel networks.

The change curves of *G*″ for MP gels, as shown in Figure 3D–F, exhibited similar trends across all groups. *G*″ showed a slow increase in the 20–50 °C range, a phase where partial denaturation of MP led to more viscous characteristics and enhanced intermolecular mobility. As the temperature gradually rose to around 52 °C, *G*″ reached its peak, during which MP molecules began to become more ordered. With a further temperature increase, *G*″ decreased. This descending phase corresponded to severe protein denaturation following the *G*″ peak, accompanied by gradual gel solidification, network stabilization and arrested viscous growth. At a constant CSO addition dosage, with increasing ultrasonic power, the peak *G*″ values generally increased compared to the U0 group. Nevertheless, *G*″ gradually dropped in the late heating stage under U3 (120 W) ultrasonic power. Conversely, with fixed ultrasonic power, elevated CSO dosage generated a more prominent rise in *G*″. The U2C2 group exhibited the highest peak *G*″ value (145.11 Pa) overall. Compared with bovine MP, native yak MP exhibits a looser conformational structure with more exposed hydrophobic amino acid residues. Ultrasonic processing may further expose hydrophobic domains of yak MP, providing abundant hydrophobic binding sites for PUFA-rich CSO. This may endow the yak MP–CSO system with a greater potential for hydrophobic interactions [20]. Based on the above rheological results, moderate ultrasound treatment combined with appropriate CSO supplementation may promote the formation of disulfide covalent cross-linking via sufficient sulfhydryl groups in MP, as well as enhance hydrophobic interactions. These synergistic effects may facilitate the construction of a uniform and dense gel network, thereby transforming the protein system from a viscoelastic liquid to a viscoelastic solid and enabling the partial formation of insoluble, compact gel structures. In contrast, excessive ultrasound treatment may impair the fabrication of a uniform and compact three-dimensional gel network.

*tan δ*, the ratio of *G*″ to *G*′, indicates a completely elastic material when approaching 0 and a completely viscous material when approaching infinity. The changes in *tan δ* values during the MP gel formation process for all groups were shown in Figure 3G–I. Overall, *tan δ* rose continuously from 30 to 45 °C, peaked at approximately 55 °C, and then sharply decreased with further temperature increase. Based on these findings, it is speculated that gel viscosity may dominate the initial heating stage of gelation, whereas elasticity may gradually become the dominant property as temperature increases. All measured *tan δ* values were below 1, indicating that elastic behavior may dominate the gel morphology. At a constant CSO addition dosage, with increasing ultrasound treatment power, *tan δ* at the end of the heating period showed an overall decreasing trend compared to the U0 group, reaching its lowest overall value at U2 (80 W). It is speculated that moderate ultrasound treatment combined with incremental CSO addition may have promoted the formation of a dense, uniform, and elastic three-dimensional gel network.

#### 3.2.2. Effects of CSO with Ultrasound on the Whiteness of MP Gels

**Figure 4:**
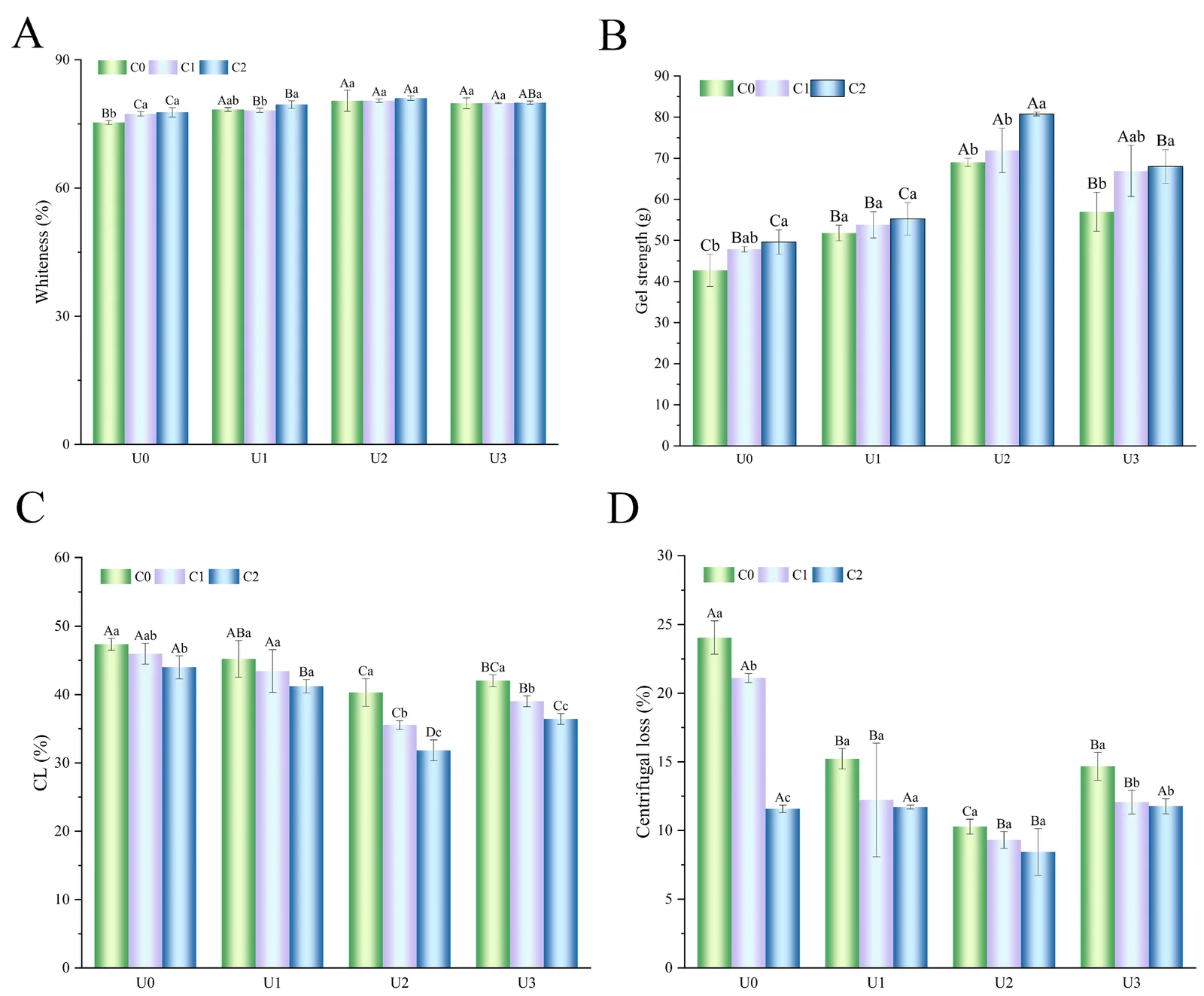
Effects of different concentrations of CSO combined with different ultrasonic power levels on the (**A**) whiteness, (**B**) gel strength, (**C**) CL, and (**D**) centrifugal loss of yak meat MP gels. Different lowercase letters above bars indicate significant differences (*p* < 0.05) among different CSO treatment groups at the same ultrasonic intensity. Different uppercase letters indicate significant differences (*p* < 0.05) among different ultrasonic intensities within the same CSO treatment group.

Whiteness can intuitively reflect the physical state of MP gels and is closely linked to changes in protein composition and the extent of protein denaturation and cross-linking. As presented in Figure 4A, with increasing ultrasonic power and CSO dosage, the whiteness of the gels in all groups showed a significant upward trend (*p* < 0.05). At a constant CSO addition dosage, as the ultrasonic power increased from U0 to U2 (0 W to 80 W), gel whiteness exhibited an increasing trend, with the optimal effect observed in the U2C2 group. Specifically, the whiteness of U2C0, U2C1, and U2C2 reached 80.40%, 80.45%, and 81.01%, respectively, representing significant increases of 6.73%, 6.79%, and 7.54% compared to their corresponding U0 groups (*p* < 0.05). It is hypothesized that ultrasound treatment may attenuate the aggregation behavior of MP, promote protein-water interactions and protein-protein cross-linking reactions, and facilitate the formation of a denser and more uniform gel network, thereby improving light scattering and reflection and consequently increasing gel whiteness. In addition, under ultrasonic action, CSO may form nano- or micron-scale emulsion droplets whose surfaces are stably coated by an adsorbed MP interfacial layer, ultimately yielding well-dispersed emulsion particles within the aqueous protein matrix. During thermal gelation, these stably and uniformly dispersed CSO nano- and micro-sized droplets may act as rigid active fillers embedded within the continuous MP gel network. Under ultrasound, they may interact with the surrounding MP matrix through hydrophobic interactions, hydrogen bonds, and covalent cross-linking—including disulfide bonds and carbonyl–amino cross-links—thereby connecting scattered protein aggregates into a more continuous, structurally dense, and well-ordered three-dimensional network. This may effectively restrict the flow and deformation of protein molecular chains, thereby enhancing the whiteness of MP gels. However, with a further increase in ultrasonic power to U3, the whiteness of gels in all U3 groups exhibited a mild, non-significant decreasing trend compared to the U2 group (*p* > 0.05). Meanwhile, under fixed U2 power, rising CSO dosage failed to significantly increase gel whiteness compared with the C0 group (*p* > 0.05), despite no obvious deterioration of gel quality. It is hypothesized that excessive ultrasound treatment may disrupt the structural stability of MP, potentially promoting MP denaturation, which may lead to the destruction of the gel network, rendering it porous and uneven and consequently reducing the whiteness of MP gels. This tendency was consistent with the foregoing results of solubility, turbidity, SDS-PAGE and rheological properties described above. Additionally, combined with the results obtained at an ultrasonic power of 120 W, this phenomenon may also be associated with the high PUFA content of CSO. CSO is susceptible to oxidation upon external stresses, which may further promote MP oxidation. Overall, appropriate CSO supplementation coupled with mild ultrasound may sustain cross-linking reactions of MP and gel-network stability. This may enable the MP gel to more effectively scatter and reflect light, synergistically enhancing gel whiteness.

#### 3.2.3. Effects of CSO with Ultrasound on the Strength of MP Gels

Gel strength serves as an important indicator to evaluate gel quality, as it objectively reflects the aggregation extent of the three-dimensional network of MP gels [48]. As illustrated in Figure 4B, with the gradual increase in ultrasonic power and CSO dosage, the gel strength exhibited an overall significant trend of first increasing and then decreasing (*p* < 0.05). At the same ultrasonic power level, gel strength increased significantly with incremental CSO addition (*p* < 0.05), showing a clear concentration dependency. Based on the above results of solubility and rheological properties, ultrasound may improve protein solubility and promote intra- and intermolecular protein interactions [49]. Meanwhile, CSO nano- or micro-droplets generated by ultrasound treatment may serve as active fillers. During heating, these droplets may bind to the protein matrix via covalent bonds or hydrophobic interactions, forming a more stable gel network and improving gel strength. At a constant CSO dosage, with increasing ultrasonic power, gel strength followed an up-then-down trend compared to the respective U0 groups. The initial increase may mainly stem from ultrasonic cavitation, which may have produced local high-temperature regions and intense shear forces, leading to MP disaggregation. This tendency was consistent with the solubility (Figure 1A) and turbidity (Figure 1B) results. Such disaggregation may have facilitated intermolecular cross-linking, promoting the formation of additional covalent bonds, hydrogen bonds, and hydrophobic interactions. These interactions further supported the assembly of a denser, more uniform gel network and improved gel strength [49]. Nevertheless, excessive ultrasonic power and CSO concentration may induce severe MP denaturation. This may have broken chemical bonds and weakened intermolecular cross-linking, ultimately decreasing gel strength. In addition, PUFAs abundant in CSO are susceptible to oxidation upon external stresses. High-power ultrasound at 120 W may trigger vigorous lipid peroxidation, which may disrupt disulfide bonds and hydrogen bonds responsible for protein structural stability and destroy α-helical structures, consequently impairing gel strength. Among all groups, U2C2 exhibited the best gel strength, which was significantly improved by 55.88% compared to the U0C0 group (*p* < 0.05). In summary, appropriate ultrasound treatment may facilitate the binding between CSO and MP, sustain protein cross-linking reactions and support the assembly of an intact gel network, thereby further enhancing or maintaining gel strength at a superior level.

#### 3.2.4. Effects of CSO with Ultrasound on the WHC of MP Gels

After heating and cooling, MP formed compact three-dimensional gel networks that tightly trapped water molecules. Therefore, the WHC of the gel acts as a crucial indicator reflecting the extent of MP denaturation and changes in spatial conformation. As displayed in Figure 4C,D, with increasing ultrasonic power and incremental CSO addition, both CL and centrifugal loss of the gels declined significantly (*p* < 0.05). At fixed ultrasonic power, gel CL and centrifugal loss decreased significantly with increasing CSO content, presenting obvious concentration dependency (*p* < 0.05). CSO may inhibit excessive protein aggregation and denaturation triggered by free radicals generated by ultrasonic cavitation, preserving native protein conformation and functionality. This protective effect may maintain intact gel networks and reduce CL and centrifugal losses, which was consistent with the results for solubility (Figure 1A) and SDS-PAGE (Figure 1C,D). At a constant CSO content, with increasing ultrasonic power, gel CL and centrifugal loss exhibited a significant decrease-then-increase trend compared to the respective U0 groups (*p* < 0.05). The U2C2 group exhibited the best WHC effect among all treatments. Its CL and centrifugal loss both significantly reduced compared to U0C0 (*p* < 0.05), decreasing by 32.73% and 65.07%, respectively. This improvement may arise because ultrasound may accelerate protein cross-linking by enhancing protein-water and intermolecular protein interactions, which may facilitate water retention within gel frameworks [50]. Such interactions may build denser, more homogeneous networks and further increase the WHC of the system. However, excessive ultrasound treatment for the U3 groups may destroy intact gel frameworks, resulting in reduced MP–water-binding capacity and a significant drop in WHC relative to the U2 groups (*p* < 0.05). Nevertheless, WHC in the U3 groups remained significantly higher than the U0 groups (*p* < 0.05), This suggests that the combined treatment may still provide favorable antioxidant effects and preserve the functional-property stability of MP.

#### 3.2.5. Effects of CSO with Ultrasound on the Water Distribution in MP Gels

**Figure 5:**
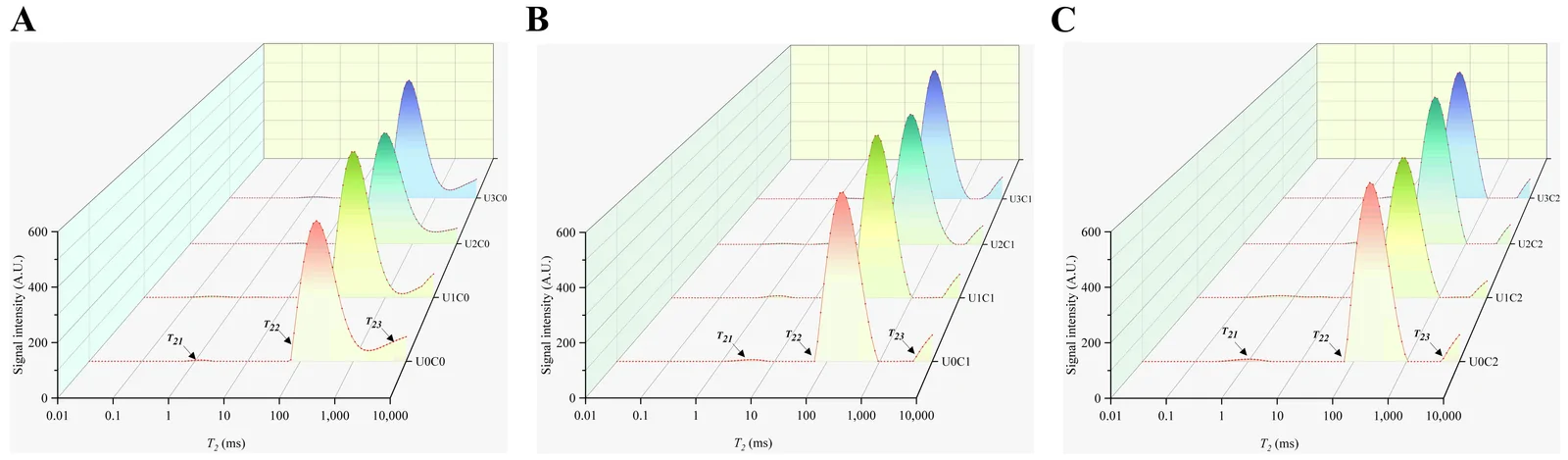
Effects of different concentrations of CSO combined with different ultrasonic power levels on the distribution of (**A**–**C**) free water, immobilized water, and bound water in yak meat MP gels, as determined by LF-NMR.

LF-NMR technology can characterize water mobility and water states within MP gels. Three distinct peaks represent three types of water fractions in MP gels. A longer *T*_2_ indicates greater water freedom and mobility. *T*_21_ (0–100 ms) represents tightly bound water associated with macromolecules. *T*_22_ (10–800 ms) represents immobilized water partially distributed within the protein network. *T*_23_ (800–1000 ms) represents free water located outside the protein network [51]. As illustrated in Figure 5A–C and Table 1, the peak area and proportion of the *T_22_* region were the largest among all groups, indicating that immobilized water dominated the water state within the MP gel network. With increasing CSO addition dosage and ultrasonic power, compared to the U0C0 group, the immobilized water content within MP gels increased, reaching the highest peak area and proportion in the U2C2 group, although the overall effect was not significant (*p* > 0.05). However, at a constant ultrasonic power, continuous CSO supplementation significantly reduced free water content in the *T*_23_ fraction relative to the corresponding C0 groups (*p* < 0.05). Concurrently, *A*_21_ showed a significant overall increasing trend compared to the C0 groups (*p* < 0.05), and the corresponding proportion *P*_21_ followed the same trend. Combined with the results from Figure 4C,D, driven by shear forces generated during ultrasonic cavitation, CSO may have interacted with MP and functioned as rigid active fillers that occupy surface voids on MP molecules. In addition, as amphiphilic components, CSO may act as interfacial mediators to enhance the water-binding capacity of MP. It may reduce free water present outside the gel matrix or transform part of this free water into bound or immobilized water, limiting water mobility and thus improving the water-holding capacity of the MP gel network [52,53]. At a constant CSO addition dosage, with increasing ultrasonic power, *A*_23_ and *P*_23_ presented a significant decrease-then-increase trend compared to the U0 group, while *A*_21_ and *P*_21_ exhibited a significant rise-then-fall tendency (*p* < 0.05). It is hypothesized that moderate ultrasound treatment may trigger mild unfolding of MP, strengthen CSO-MP interactions and MP–water retention, reduce the proportion of free water and increase bound-water fractions. These alterations may improve the WHC of MP gels and stabilize gel microstructures. However, with a further increase in ultrasonic power to U3 (120 W) compared to the U2 group (80 W), *A*_23_ and *P*_23_ showed an increasing trend, while *A*_21_ and *P*_21_ showed an overall decreasing trend, which corresponded to the WHC results mentioned earlier. This may stem from cavitation effects and mechanical forces produced by high-power ultrasound, which may have disrupted the stable MP gel-network structure, induced excessive MP unfolding and accumulation of disordered structures and ultimately caused protein denaturation. Such structural impairment may reduce the water-binding capacity of MP and transform immobilized water into free water. The consequent water loss may have further increased the content of highly mobile free water inside the MP matrix. In summary, the combined ultrasound and CSO treatment may exert no significant influence on immobilized water content in MP gels (*p* > 0.05). Nevertheless, CSO supplementation significantly strengthened MP’s ability to bind bound water and altered the proportion of free water (*p* < 0.05). Moreover, moderate ultrasonic power also exhibited a synergistic role in water fixation overall, significantly reducing the proportion of free water within MP gels (*p* < 0.05), thereby promoting the WHC and stability of the MP gel network.

#### 3.2.6. Effects of CSO with Ultrasound on the Textural Properties of the Gels

**Figure 6:**
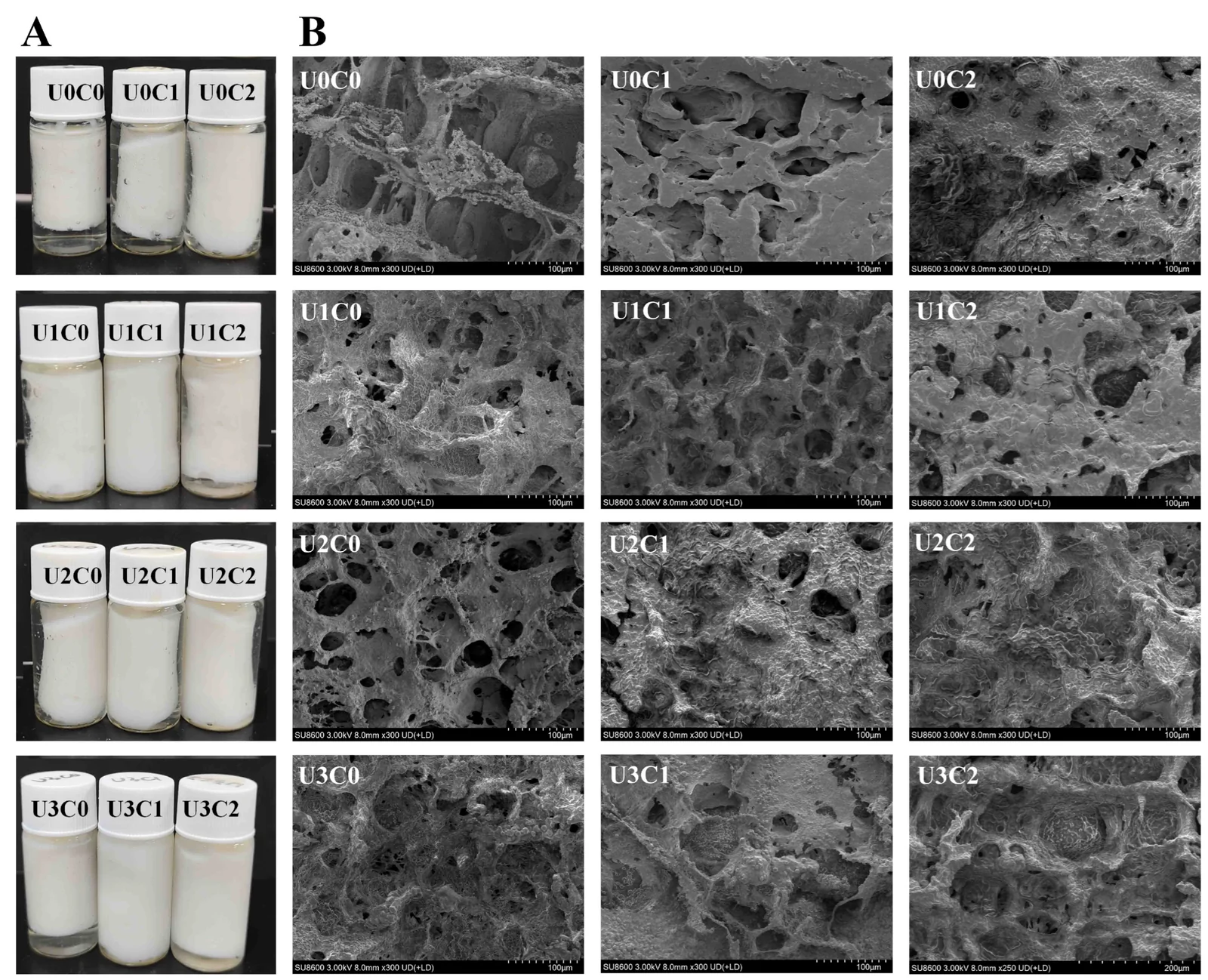
Effects of different concentrations of CSO combined with different ultrasonic power levels on the (**A**) macroscopic morphology and (**B**) microstructure (SEM images) of yak meat MP gels.

Figure 6A,B illustrates the effects of varying ultrasonic powers combined with varying CSO addition dosages on the macroscopic appearance and microstructure of MP after thermal gelation. As displayed in Figure 6A, the organizational states of the MP gels differed among groups after heat induction. Furthermore, as observed in Figure 6B, under the possible constraints imposed by the intrinsic properties of CSO, pores of varying sizes were observed on the surface of all gel samples. Overall, at a fixed ultrasonic power (U0–U2), increasing CSO addition reduced the number and size of pores compared with the group without essential oil addition (C0). Meanwhile, at a fixed CSO concentration, increasing ultrasonic powers (U0–U2) progressively diminished the sheet-like structures, and the large-void structures gradually transformed into small, uniform pores and filamentous structures. Previous studies have reported that ultrasound treatment during heat-induced gelation facilitates the formation of a well-structured, uniform, and dense gel network [49]. In conjunction with the results presented in Figure 2, Figure 3, Figure 4 and Figure 5, it is hypothesized that the incorporation of CSO may effectively fill the voids within the gel network and, when combined with ultrasound treatment, may contribute to the formation of a more uniform elastic MP gel and enhanced WHC. However, the optimal treatment conditions for improving the MP gel network structure could not be determined under the present conditions, primarily because, for the C1 and C2 CSO groups, the pores observed in the corresponding images may have arisen from lipid loss of CSO. Nevertheless, compared with the U0C0 group (without ultrasound and without essential oil), which exhibited large pores, abundant sheet-like structures, and a rough surface, the combination of CSO and ultrasound treatment holds considerable promise for improving the MP gel structure. When the ultrasonic power was further increased to U3 (120 W), examination of the gel’s organizational state and microstructure under the constraints imposed by the intrinsic properties of CSO revealed that, relative to the U2 group, the gel tended to become looser, with an increase in sheet-like structures and a rougher surface. It is hypothesized that higher ultrasonic power may exert adverse effects on the MP gel structure, leading to the formation of irregular pores [54]; however, considering the intrinsic properties of CSO, these pores may also result from residual voids left by the loss of small amounts of CSO droplets.

#### 3.2.7. Correlation and Mechanism Analysis

**Figure 7:**
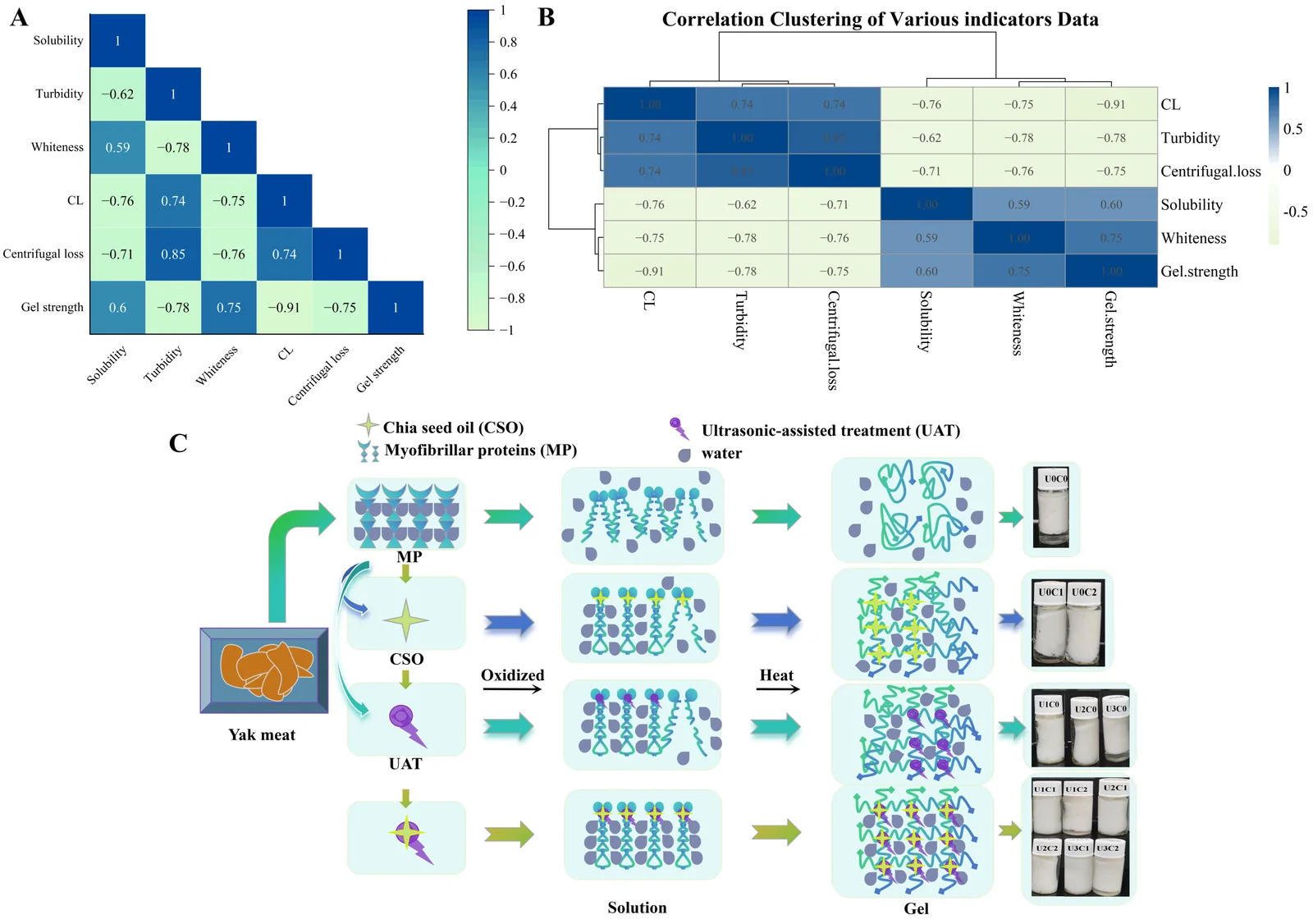
Panels (**A**,**B**): Correlation analysis among various indicators of yak meat MP and its gels treated with different concentrations of CSO and ultrasonic power. Panel (**C**): Schematic diagram illustrating the proposed mechanism for the improvement of yak meat MP functional properties by the combined treatment of CSO and ultrasound.

Pearson correlation analysis was conducted, and the results presented in Figure 7A,B revealed hierarchical clustering relationships among all detection indicators. Based on similarity, the six indicators were clustered into three major groups. Gel strength, solubility, and whiteness formed one functional cluster, while centrifugal loss, turbidity, and CL formed another, collectively characterizing the structural and gel functional properties of MP. Pairwise correlations between all indicators reached moderate or higher levels (|*r*| ≥ 0.5), demonstrating strong interrelation among these measurement indices. For instance, CL exhibited a strong positive correlation with turbidity and centrifugal loss (*r* = 0.74), an extremely strong negative correlation with gel strength (*r* = −0.91), and strong negative correlations with solubility and whiteness (*r* = −0.76, *r* = −0.75, respectively). Centrifugal loss exhibited a strong negative correlation with whiteness (*r* = −0.76), etc. The clustering of centrifugal loss, turbidity, and CL corresponded to a dispersed state of the MP gel system, whereas their opposite state indicates a cohesive state. The clustering of gel strength, solubility, and whiteness suggested that better system dissolution corresponded to higher whiteness and stronger gel strength, characterizing the solubility, appearance/color, and WHC of MP. The extremely strong negative correlation between gel strength and CL indicated that stronger gel strength corresponded to smaller CL, signifying better WHC and gel texture.

Figure 7C illustrates the potential mechanism by which CSO combined with moderate ultrasound treatment improves the structural and gel functional properties of yak MP under the experimental conditions used in this study. The cavitation effect generated by ultrasound may optimize MP component composition and increase MP hydrophilicity and interfacial tension. Meanwhile, ultrasound produces nano- and microscale CSO droplets that may act as active fillers via synergistic interactions with MP. During thermal gelation, these droplets may suppress excessive protein self-aggregation and construct stable gel networks. Meanwhile, CSO may protect the native conformation and function of MP, ultimately improving structural characteristics including solubility and turbidity. Furthermore, ultrasonic mechanical force may rearrange the spatial conformation of MP and strengthen MP-CSO interactions. This process facilitates moderate disulfide cross-linking and promotes the formation of stable covalent bonds, hydrogen bonds, and hydrophobic non-covalent interactions. These interactions may drive the migration of low-molecular-weight proteins to assemble into stable macromolecular myosin heavy chains (*MHC*, ~180 kDa). This assembly may reduce chromatographic tailing originating from the cross-linking of sulfhydryl-rich actin and generate denser, highly ordered, homogeneous elastic gel networks. Moreover, abundant lipid fractions in CSO may strengthen protein-water binding under ultrasonic mechanical stimulation. Consequently, the system retains more water within MP frameworks, reduces free water content and water molecular mobility, and greatly improves the gel WHC.

## 4. Conclusions

Under the experimental conditions for this set of mixed meat samples, the present study demonstrated that moderate ultrasound treatment combined with CSO exerted a synergistic effect, significantly improving the structural and gel-functional properties of MP, with the overall optimal effect observed in the U2C2 group (80 W, 3.0%). However, in this study, the application of excessively high ultrasonic power (120 W) in combination with CSO may adversely affect MP structure and gel functional properties. Therefore, this necessitates the optimization of relevant parameters in subsequent pilot-scale trials. Furthermore, this study focused primarily on the physicochemical properties of ultrasound-assisted CSO treatment of MP gels, without evaluating their oxidative stability during long-term storage. Therefore, future research will center on dynamic oxidation indicators of MP under simulated commercial refrigeration conditions, such as conventional chilling or superchilling preservation, to investigate the effects of ultrasound-assisted CSO on MP oxidation characteristics. In addition, methodological improvements will be made to assess the microstructural effects of CSO ultrasound on MP gel properties. Specifically, osmium tetroxide will be employed to fix the lipid components of CSO in the experiment, thereby eliminating potential artifacts caused by lipid droplet loss and enabling a more accurate determination of the effects of ultrasound–CSO on the microstructure of MP gels. In summary, under the conditions of the mixed meat samples employed in this study, moderate ultrasound combined with CSO treatment improved the functional properties of meat products, and can be used to enhance the textural properties and WHC of yak MP. This study provides a preliminary theoretical basis for pilot-scale trials of CSO combined with ultrasound treatment in the development of healthy, high-quality yak meat products that meet clean-label requirements.

## Figures and Tables

**Table 1:** Effects of different concentrations of CSO combined with different ultrasonic power levels on the peak areas (*A*_21_, *A*_22_, *A*_23_) and proportions (*P*_21_, *P*_22_, *P*_23_) of bound water, immobilized water, and free water in yak meat MP gels. Different lowercase letters indicate significant differences (*p* < 0.05) among different CSO treatment groups at the same ultrasonic intensity. Different uppercase letters indicate significant differences (*p* < 0.05) among different ultrasonic intensities within the same CSO treatment group.

	*A*_21_	*A*_22_	*A*_23_	*P*_21_	*P*_22_	*P*_23_
U0C0	27.3 ± 2.0 ^Cb^	6618.4 ± 810.6 ^Aa^	794.7 ± 97.3 ^Aa^	0.40 ± 0.05 ^Cb^	88.9 ± 10.9 ^Aa^	10.7 ± 1.3 ^Aa^
U1C0	54.7 ± 6.7 ^Ba^	7275.0 ± 891.0 ^Aa^	498.2 ± 63.6 ^Ab^	0.70 ± 0.09 ^Ba^	92.6 ± 12.3 ^Aa^	6.7 ± 0.8 ^Ab^
U2C0	12.1 ± 1.5 ^Bc^	6783.9 ± 830.8 ^Aa^	485.0 ± 59.4 ^Ab^	0.17 ± 0.02 ^Bc^	93.2 ± 15.4 ^Aa^	6.7 ± 0.4 ^Ab^
U3C0	46.92 ± 5.75 ^Aa^	7000.6 ± 857.4 ^Aa^	690.0 ± 84.5 ^Aa^	0.61 ± 0.07 ^Aa^	90.5 ± 11.1 ^Aa^	8.9 ± 1.1 ^Aa^
U0C1	55.6 ± 6.8 ^Bb^	7250.6 ± 888.0 ^Aa^	374.8 ± 45.9 ^Bab^	0.72 ± 0.09 ^Bb^	94.4 ± 12.1 ^Aa^	4.9 ± 0.6 ^Bab^
U1C1	66.2 ± 8.1 ^Bab^	7627.3 ± 934.1 ^Aa^	365.6 ± 44.8 ^Bab^	0.82 ± 0.10 ^Bab^	94.6 ± 11.7 ^Aa^	4.5 ± 0.6 ^Bb^
U2C1	71.4 ± 8.8 ^Aa^	7286.7 ± 892.4 ^Aa^	328.3 ± 40.2 ^Bb^	0.93 ± 0.11 ^Aa^	94.8 ± 13.6 ^Aa^	4.3 ± 0.5 ^Bb^
U3C1	29.0 ± 3.6 ^Bc^	7255.8 ± 868.6 ^Aa^	448.6 ± 55.0 ^Ba^	0.38 ± 0.05 ^Bc^	93.8 ± 11.5 ^Aa^	5.8 ± 0.7 ^Ba^
U0C2	88.4 ± 10.8 ^Ab^	7503.4 ± 919.0 ^Aa^	352.2 ± 43.1 ^Ba^	1.11 ± 0.14 ^Ab^	94.5 ± 13.6 ^Aa^	4.4 ± 0.5 ^Ba^
U1C2	133.6 ± 16.3 ^Aa^	6807.83 ± 833.8 ^Aa^	259.6 ± 31.8 ^Cb^	1.87 ± 0.23 ^Aa^	94.5 ± 14.3 ^Aa^	3.6 ± 0.4 ^Ba^
U2C2	64.8 ± 7.9 ^Ac^	7727.4 ± 946.4 ^Aa^	277.0 ± 33.9 ^Bab^	0.80 ± 0.10 ^Ac^	95.8 ± 11.7 ^Aa^	3.4 ± 0.4 ^Ba^
U3C2	35.5 ± 4.4 ^Bd^	7017.5 ± 860.6 ^Aa^	299.7 ± 36.7 ^Cab^	0.48 ± 0.06 ^Bd^	95.4 ± 10.7 ^Aa^	4.1 ± 0.5 ^Ca^

## Data Availability

The original contributions presented in this study are included in the article. Further inquiries can be directed to the corresponding authors.

## Abbreviations

The following abbreviations are used in this manuscript:

CSO	chia seed oil
MP	myofibrillar protein
SDS-PAGE	sodium dodecyl sulfate polyacrylamide gel electrophoresis
MHC	myosin heavy chain
CL	cooking loss (%)
WHC	water-holding capacity (%)
PUFAs	polyunsaturated fatty acids
PBS	phosphate-buffered saline
*L**, *a**, *b**	the color parameters
*m*_1_	the mass of the MP solution before heating (g)
*m*_2_	the mass of the MP solution after heating (g)
*m*_0_	the masses of the empty tube (g)
*T*	transverse relaxation time (ms)
*A*_2_	peak areas
*P*_2_	peak proportional areas
C0, C1, C2	chia seed oil concentration parameters (*w*/*w*)
U0, U1, U2, U3	ultrasonic power parameters (W)
U0C0, U0C1, U0C2; U1C0, U1C1, U1C2; U2C0, U2C1, U2C2; U3C0, U3C1, U3C2	chia seed oil combined with ultrasonic power parameters
*G*′	storage modulus (Pa)
*G*″	loss modulus (Pa)
*tan δ*	loss tangent (Pa)
*T*_21_	tightly bound water (A.U.)
*T*_22_	immobilized water (A.U.)
*T*_23_	free water (A.U.)
*r*	Pearson correlation coefficient
